# Exploring miRNA–target gene pair detection in disease with coRmiT

**DOI:** 10.1093/bib/bbae060

**Published:** 2024-03-02

**Authors:** Jose Cordoba-Caballero, James R Perkins, Federico García-Criado, Diana Gallego, Alicia Navarro-Sánchez, Mireia Moreno-Estellés, Concepción Garcés, Fernando Bonet, Carlos Romá-Mateo, Rocio Toro, Belén Perez, Pascual Sanz, Matthias Kohl, Elena Rojano, Pedro Seoane, Juan A G Ranea

**Affiliations:** Departamento de Biología Molecular y Bioquímica, Facultad de Ciencias, Universidad de Málaga, Bulevar Louis Pasteur, 31, Málaga, 29010, Spain; Research Unit, Biomedical Research and Innovation Institute of Cádiz (INiBICA), Puerta del Mar University Hospital, Cádiz, Spain; Departamento de Biología Molecular y Bioquímica, Facultad de Ciencias, Universidad de Málaga, Bulevar Louis Pasteur, 31, Málaga, 29010, Spain; Instituto de Investigación Biomédica de Málaga y Plataforma en Nanomedicina (IBIMA-Plataforma BIONAND), C/ Severo Ochoa, 35, Parque Tecnológico de Andalucía (PTA), Campanillas, Málaga, 29590, Spain; Departamento de Biología Molecular y Bioquímica, Facultad de Ciencias, Universidad de Málaga, Bulevar Louis Pasteur, 31, Málaga, 29010, Spain; CIBER de Enfermedades Raras (CIBERER), Avda. Monforte de Lemos, 3-5, Pabellón 11, Planta 0, Madrid, 28029, Spain; Centro de Diagnóstico de Enfermedades Moleculares, Centro de Biología Molecular-SO UAM-CSIC, Universidad Autónoma de Madrid, Campus de Cantoblanco, Madrid, Spain; Instituto de Investigación Sanitaria IdiPaZ, Madrid, Spain; CIBER de Enfermedades Raras (CIBERER), Avda. Monforte de Lemos, 3-5, Pabellón 11, Planta 0, Madrid, 28029, Spain; Departament de Fisiologia, Facultat de Medicina i Odontologia, Universitat de València, Av. Blasco Ibáñez 15, 46010, València, Spain; CIBER de Enfermedades Raras (CIBERER), Avda. Monforte de Lemos, 3-5, Pabellón 11, Planta 0, Madrid, 28029, Spain; Consejo Superior de Investigaciones Científicas, Instituto de Biomedicina de Valencia, Jaime Roig 11, 46010, Valencia, Spain; CIBER de Enfermedades Raras (CIBERER), Avda. Monforte de Lemos, 3-5, Pabellón 11, Planta 0, Madrid, 28029, Spain; Departament de Fisiologia, Facultat de Medicina i Odontologia, Universitat de València, Av. Blasco Ibáñez 15, 46010, València, Spain; Research Unit, Biomedical Research and Innovation Institute of Cádiz (INiBICA), Puerta del Mar University Hospital, Cádiz, Spain; Medicine Department, School of Medicine, University of Cádiz, Cádiz, Spain; CIBER de Enfermedades Raras (CIBERER), Avda. Monforte de Lemos, 3-5, Pabellón 11, Planta 0, Madrid, 28029, Spain; Departament de Fisiologia, Facultat de Medicina i Odontologia, Universitat de València, Av. Blasco Ibáñez 15, 46010, València, Spain; Incliva Biomedical Research Institute, 46010, València, Spain; Research Unit, Biomedical Research and Innovation Institute of Cádiz (INiBICA), Puerta del Mar University Hospital, Cádiz, Spain; Medicine Department, School of Medicine, University of Cádiz, Cádiz, Spain; CIBER de Enfermedades Raras (CIBERER), Avda. Monforte de Lemos, 3-5, Pabellón 11, Planta 0, Madrid, 28029, Spain; Centro de Diagnóstico de Enfermedades Moleculares, Centro de Biología Molecular-SO UAM-CSIC, Universidad Autónoma de Madrid, Campus de Cantoblanco, Madrid, Spain; Instituto de Investigación Sanitaria IdiPaZ, Madrid, Spain; CIBER de Enfermedades Raras (CIBERER), Avda. Monforte de Lemos, 3-5, Pabellón 11, Planta 0, Madrid, 28029, Spain; Consejo Superior de Investigaciones Científicas, Instituto de Biomedicina de Valencia, Jaime Roig 11, 46010, Valencia, Spain; Faculty of Medical and Life Sciences, Furtwangen University, Germany; Departamento de Biología Molecular y Bioquímica, Facultad de Ciencias, Universidad de Málaga, Bulevar Louis Pasteur, 31, Málaga, 29010, Spain; Instituto de Investigación Biomédica de Málaga y Plataforma en Nanomedicina (IBIMA-Plataforma BIONAND), C/ Severo Ochoa, 35, Parque Tecnológico de Andalucía (PTA), Campanillas, Málaga, 29590, Spain; Departamento de Biología Molecular y Bioquímica, Facultad de Ciencias, Universidad de Málaga, Bulevar Louis Pasteur, 31, Málaga, 29010, Spain; Instituto de Investigación Biomédica de Málaga y Plataforma en Nanomedicina (IBIMA-Plataforma BIONAND), C/ Severo Ochoa, 35, Parque Tecnológico de Andalucía (PTA), Campanillas, Málaga, 29590, Spain; CIBER de Enfermedades Raras (CIBERER), Avda. Monforte de Lemos, 3-5, Pabellón 11, Planta 0, Madrid, 28029, Spain; Departamento de Biología Molecular y Bioquímica, Facultad de Ciencias, Universidad de Málaga, Bulevar Louis Pasteur, 31, Málaga, 29010, Spain; Instituto de Investigación Biomédica de Málaga y Plataforma en Nanomedicina (IBIMA-Plataforma BIONAND), C/ Severo Ochoa, 35, Parque Tecnológico de Andalucía (PTA), Campanillas, Málaga, 29590, Spain; CIBER de Enfermedades Raras (CIBERER), Avda. Monforte de Lemos, 3-5, Pabellón 11, Planta 0, Madrid, 28029, Spain; Instituto Nacional de Bioinformática (INB/ELIXIR-ES), Instituto de Salud Carlos III (ISCIII), C/ Sinesio Delgado, 4, Madrid, 28029, Spain

**Keywords:** miRNA, RNA-Seq, correlation, odds ratio, genetic disease, target

## Abstract

A wide range of approaches can be used to detect micro RNA (miRNA)–target gene pairs (mTPs) from expression data, differing in the ways the gene and miRNA expression profiles are calculated, combined and correlated. However, there is no clear consensus on which is the best approach across all datasets. Here, we have implemented multiple strategies and applied them to three distinct rare disease datasets that comprise smallRNA-Seq and RNA-Seq data obtained from the same samples, obtaining mTPs related to the disease pathology. All datasets were preprocessed using a standardized, freely available computational workflow, DEG_workflow. This workflow includes coRmiT, a method to compare multiple strategies for mTP detection. We used it to investigate the overlap of the detected mTPs with predicted and validated mTPs from 11 different databases. Results show that there is no clear best strategy for mTP detection applicable to all situations. We therefore propose the integration of the results of the different strategies by selecting the one with the highest odds ratio for each miRNA, as the optimal way to integrate the results. We applied this selection-integration method to the datasets and showed it to be robust to changes in the predicted and validated mTP databases. Our findings have important implications for miRNA analysis. coRmiT is implemented as part of the ExpHunterSuite Bioconductor package available from https://bioconductor.org/packages/ExpHunterSuite.

## INTRODUCTION

MicroRNAs (miRNAs) are small RNA sequences of $\sim $22 nt that have an important role in post-transcriptional repression and regulation of mRNA expression in many biological systems. Their research is important for disease as they show potential as biomarkers and therapeutic targets [1, 2].

Transcriptomic sequencing technologies are increasingly being applied to various aspects of gene expression. An important approach is to integrate *in silico* the expression of mRNAs and miRNAs to detect miRNA–target gene pairs (mTPs).

The simplest strategy to find mTPs is to analyze the expression of mRNA and miRNA separately to find differentially expressed genes (DEGs) and differentially expressed miRNAs (DEMs), and then determine which DEGs–DEMs pairs are likely to be mTPs using sequence based prediction methods [3–5]. Such methods include TargetScan [6], PITA [7], miRanda [8] and miRDB [9]. However, only TargetScan and miRDB have been consistently updated over the last 8 years [10] and the precision of recently published predictive tools is still low when compared with high-throughput validation datasets [9, 11, 12]. In recent years, new tools based on deep learning have emerged, such as DMISO [13], TargetNet [14], miRBind [15] and ncRNAInter [16].

Anti-correlation between the expression levels of miRNAs and their targets also provides evidence of potential regulation. Many studies have exploited this idea to find mTPs [17–20]. However, there is considerable difference in the implementation of this general approach between studies. Some have calculated the Pearson correlation coefficient between all miRNA and mRNA expression profiles directly [17, 18]. Others have grouped the mRNAs into co-expression modules using weighted gene correlation network analysis (WGCNA) as a first step, then calculated the correlation coefficient between co-expressed genes and the single miRNA expression profiles using the module eigengene to represent the gene modules [19]. In other studies they have grouped miRNAs and mRNAs, respectively, into co-expression modules and computed the correlation coefficient between their module eigengenes [20, 21].

In addition, there are studies that filter the detected mTPs using databases of experimentally validated mTPs [22]. Such databases include miRTarBase [23], Tarbase [24] and miRecords [25].

Despite there being multiple approaches for mTP detection based on anti-correlation, there is no clear consensus as to which leads to the most reliable results.

Here we present coRmiT, a novel method for the analysis, comparison, selection and integration of seven mTP correlation strategies based on mRNA and miRNA expression data, using a range of anti-correlation thresholds. We apply it to three distinct datasets with very different properties, all of which model rare diseases. We evaluate the strategies by analyzing the mTPs detected by each approach at different correlation thresholds, comparing them with predicted and experimentally validated mTPs sourced from databases. Finally, we propose a novel method that integrates the results of the correlation strategies at the miRNA level and demonstrate its robustness. coRmiT is available as part of the ExpHunterSuite Bioconductor package.

## MATERIAL AND METHODS

In this study, we implemented coRmiT to investigate different strategies for detecting mTPs by comparing expression profiles, and applied it to multiple datasets.

As coRmiT implements multiple strategies to find mTPs, we performed a systematic comparison of the results derived from these strategies, evaluating their overlap with both predicted and experimentally validated mTPs. We also implemented a novel approach based on selecting the best-performing strategy at the miRNA level and integrating the results. The coRmiT results for the three different datasets were used for functional analysis and a selection of genes were validated in terms of expression.

### Experimental datasets

For each experiment, data were generated in the form of miRNA and mRNA sequence data (fastq files) using Illumina technology. The data and experimental models are described as follows:

**PMM2 congenital disorder of glycosylation**

PMM2 congenital disorder of glycosylation (PMM2-CDG) is caused by loss-of-function mutations affecting the PMM2 enzyme [26, 27], leading to impaired protein glycosylation. Currently, there is no effective treatment available [26]. The dataset used in this study consists of skin fibroblast cell lines, including five derived from PMM2-CDG patients and five from healthy individuals. The patients were considered to have a high degree of disease severity, determined according to the Nijmegen Pediatric CDG Rating Score (NPCRS), the International Cooperative Ataxia Rating Scale (ICARS) and the midsaggital relative vermis diameter (MVRD) based on magnetic resonance imaging. Libraries were prepared using the TruSeq Stranded mRNA Library Prep Kit (Illumina, San Diego, CA) and sequenced in a NovaSeq 6000 system (Illumina, San Diego, CA). This RNA-Seq dataset consists of 100 bp paired end reads, with an average depth of 37.7 M reads. The smallRNA-Seq libraries were produced using the TruSeq Small RNA Library Preparation Kit and sequenced in a NextSeq500 platform (Illumina, San Diego, CA). The initial smallRNA-Seq dataset consists of 75 bp single end reads, with an average depth of 16.0 M reads per sample.

**Lafora Disease**

Lafora disease (LD) is a neurodegenerative disorder characterized by the inclusion of insoluble poorly branched glycogen, forming Lafora bodies within neurons. This dataset was designed to compare the transcriptomic expression of four wild-type mice and seven knock out mice mutants for the *Epm2a* (three mice) and *Epm2b* (four mice) genes in brain. The smallRNA-Seq libraries were generated using the NEXTFLEX small RNA-Seq kit v3 (Perkin Elmer, Waltham, MA, USA). The cDNA libraries were generated using the TruSeq Stranded mRNA LP kit (48 Spl) (Illumina, San Diego, CA). Both smallRNA-Seq and RNA-Seq samples were sequenced using Illumina NextSeq 550, obtaining reads of 75 and 50 bp for RNA-Seq and smallRNA-Seq, respectively. The average depth was 18.0 M reads for the RNA-Seq samples and 5.6 M reads for the smallRNA-Seq samples. Further sequencing details for the RNA-Seq experiment are given in [28, 29] (mRNA) and [30] (miRNA).

**Dilated cardiomyopathy**

Dilated cardiomyopathy (DCM) encompasses a set of heart diseases characterized by the presence of left ventricular or biventricular dilatation and systolic dysfunction. Mutations in *LMNA* can cause DCM [31]. This dataset was designed to compare myocardial samples of six wild-type mice and six *Lmna* mutant mice with DCM. The mRNA-Seq data were produced using the NEBNext Ultra II Directional RNA Library Prep Kit for Illumina, using the NEBNext Poly(A) mRNA Magnetic Isolation Module (New England Biolabs, Ipswich, MA). Libraries were sequenced on an Illumina PE75 Platform with an average depth of 70.7 M reads of 72 bp. The smallRNA-Seq samples were generated using the NEXTFLEX small RNA-Seq kit v3 (Perkin Elmer, Waltham, MA, USA), and single-end libraries were sequenced on an Illumina SE75 Platform, producing 75 bp reads, with an average depth of 22.1 M reads.

### Expression analysis workflow

We obtained normalized expression tables, lists of DEGs/DEMsand modules of co-expressed genes/miRNAs from fastq files using an automated workflow for miRNA-Seq/RNA-Seq expression analysis. This workflow was implemented with the AutoFlow workflow manager [32]. Full details are shown in Supplementary Methods (Supplementary Figure 1). This workflow comprises (i) an miRNA detection module that identifies the miRNA sequences in all fastq files; (ii) an miRNA quantification module that counts the reads that correspond to each identified miRNA from the miRNA detection module and quantifies their expression and (iii) an RNA-Seq analysis module that quantifies genes and analyzes their expression. The workflow is available at https://github.com/seoanezonjic/DEG_workflow.

### Correlation strategies to obtain mTPs: coRmiT

**Figure 1:**
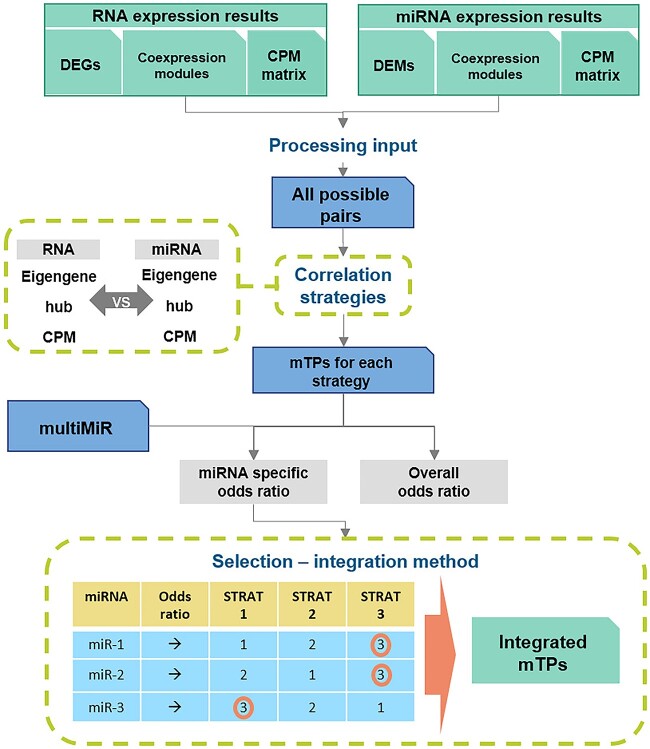
General overview of the coRmiT methodology. Sequencing data for miRNA and gene expression are processed to generate all possible pairs of expressed genes/miRNAs. Correlation between these pairs is computed using different strategies that combine individual gene/miRNA expression and co-expression module expression. Odds ratios are computed for each strategy (Overall odds ratio) and each miRNA (Specific odds ratio) based on overlap with mTPs obtained from databases. The selection-integration method ranks, for each miRNA, the strategies based on their specific odds ratio and selects the top strategy. The integrated mTPs are those detected with the selected strategy for each miRNA. **DEGs** and **DEMs** represent the differentially expressed genes and miRNAs, respectively. **CPM** is the expression matrix normalized to show counts per million mapped reads for each gene/miRNA. **Eigengene** and **hub** are the representative profiles for each co-expression module. **mTPs** are the miRNA–target gene pairs. **STRAT N** represents an example correlation strategy and **miR-N** represents an example miRNA.

Multiple approaches have been used to obtain mTPs from expression data based on anti-correlation [17–21]. We have designed coRmiT to implement different strategies, rather than focusing on a single approach, based on the expression of individual genes and miRNAs, and co-expression modules. Multiple correlation thresholds have been used for each strategy, from -0.9 to -0.5 in intervals of 0.05. Overall and specific odds ratios are computed for the different strategies and correlation thresholds, to quantify their overlap with mTPs obtained from databases. Strategies are then combined using the selection-integration method. This method ranks the strategies for each miRNA based on the specific odds ratio and selects the mTPs for the top strategy (Figure 1). Full details are given in Supplementary Methods.

### Testing the robustness of the selection-integration method rankings

The selection-integration method generates a ranking of strategies and correlation thresholds for each miRNA based on the odds ratios. To assess the robustness of this ranking in response to changes in the underlying databases, we re-ranked the strategies and thresholds using randomized subsets of the multiMiR mTPs.

This process involved randomly selecting 75% of the multiMiR mTPs and recalculating the miRNA specific odds ratios, following the procedure described above, for each strategy and correlation threshold. This random sampling was repeated 40 times, and the resulting rankings were compared with the ranking obtained using the complete multiMiR mTPs dataset.

### Functional enrichment of miRNA targets

Over-representation analysis was conducted for the targets of each miRNA using the Gene Ontology (GO) [33], KEGG [34] and Reactome [35]. We used the ExpHunterSuite script *clusters_to_enrichment.R*, which is based on the clusterProfiler package [36], to perform the analysis. An FDR threshold (*-p 0.1*) was applied, and the parental GO terms of each significantly enriched term were removed from the results using the argument *-c*.

### Analyzing positive correlation with coRmiT

We also used coRmiT to inspect the positive correlations between miRNAs and their targets in the LD dataset. We varied the correlation thresholds from 0.5 to 0.9 in intervals of 0.05. With these thresholds and the *–corr_type higher* option, coRmiT identified pairs with a Pearson correlation exceeding the thresholds, which were considered strategy mTPs. Subsequently, we computed the specific miRNA odds ratio and performed Fisher’s exact test for each strategy and correlation threshold combination. The selection-integration method was applied in the same way as for the anti-correlated mTPs.

### Validation of target gene expression changes

The expression levels of the putative LD targets were analyzed by RT-qPCR to confirm differential expression. The target genes selected were *Tert*, *Tgm1*, *Trem2*, *Smc1A*, *Gabrg2*, *Gabrb3*, *Gfap*, *Tyrobp*, *Arg1* and *Psmb8*. Further details of the RT-qPCR procedure and gene target selection can be found in Supplementary Methods, in Section: ‘Validation of differential expression of targets in LD’.

## RESULTS

For each dataset, the expression data were analyzed to obtain the CPM matrix, co-expression modules and DEGs and DEMs, all three of which are necessary to run coRmiT. A summary of the expression analysis results is shown in Table 1. Additional results are available in the Online Repository (https://github.com/JoseCorCab/coRmiT_additional_files). Full details of each analysis are shown in the Online Repository Files 1, 2 and 3.

**Table 1:** Summary of the expression analysis results for the three datasets. The DEGs column shows the numbers of differentially expressed genes; the DEMs column shows the differentially expressed miRNAs. The DE packages column shows the differential expression packages used to find DEMs. The modules column indicates the number of gene modules found by WGCNA. DCM: dilated cardiomyopathy, PMM2-CDG: PMM2 congenital disorder of glycosylation, LD: Lafora Disease, L: limma, D: DESeq2 and E: edgeR

Project	DEGs	Gene modules	DEMs	DE Packages	miRNA modules
DCM	2148	60	53	E,D	7
PMM2-CDG	415	163	17	E,D,L	55
LD	179	350	3	E,D	15

For the DCM dataset, we observed more than five times as many DEGs and three times as many DEMs compared with the other datasets. However, genes and miRNAs were distributed across fewer WCGNA modules. In terms of the DEM detection methods, NOISeq did not yield any significant results, and limma only identified significant results for the PMM2-CDG dataset.

Using the filtered data, coRmiT was used to detect anti-correlated mTPs, using the seven strategies shown in the Supplementary Methods, Table 1. For each strategy, an overall odds ratio was calculated for each of the different correlation thresholds investigated. This calculation was based on the overlap between the mTPs detected for each strategy with correlation values lower than the threshold and the predicted/validated mTPs obtained from multiMiR, as described in the Methods section.

### No consensus found for the best strategy or threshold

**Figure 2:**
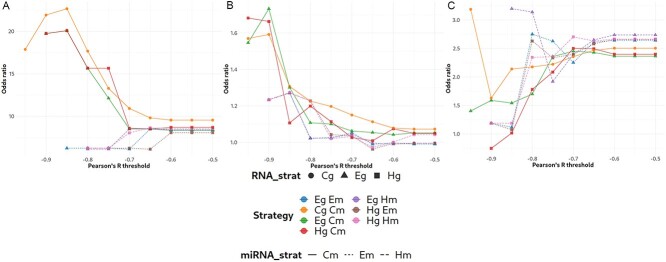
Overall odds ratios of all strategies for the **A** LD, **B** DCM and **C** PMM2-CDG datasets. Overall odds ratios are shown on the Y-axis; different correlation thresholds are shown on the X-axis. Colors, points and line styles are used to indicate the different strategies.

The overall odds ratio values for all strategy and correlation threshold combinations are shown in Figure 2. For the LD and DCM datasets, the three strategies that led to the highest odds ratio values at more restrictive correlation thresholds were those that compare single miRNA expression profiles with the different gene expression profiles (Figures 2A and B). Conversely, strategies that correlate the representative profiles for the miRNAs with gene modules performed better for the PMM2-CDG dataset (Figure 2C), except for very strict correlation thresholds where the gene count, miRNA count strategy performed best. However, this led to a comparatively small number of detected mTPs (20, of which five are multiMiR mTPs).

Focusing on the miRNA count expression profile methods, when comparing odds ratio values at different correlation thresholds for the different strategies, we see that for the LD and DCM datasets, more restrictive thresholds tended to lead to higher odds ratio values, with there being a critical correlation value, above which the odds ratios tended to decrease rapidly; this critical value was different for each dataset: -0.85 and -0.9, respectively, for LD and DCM. The rate of the decrease in odds ratio values also differed between these datasets. Conversely, the PMM2-CDG dataset actually showed a gradual increase in odds ratios with more restrictive thresholds for most strategies (Figure 2C).

In general, although the LD and DCM datasets showed some similarity, there was no strategy and correlation threshold combination that performed best for all datasets.

### Strategies perform differently at the miRNA level

**Figure 3:**
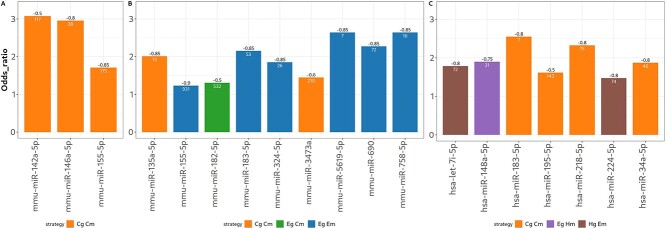
Top strategy and correlation thresholds according to the selection-integration method for the **A** LD, **B** DCM and **C** PMM2-CDG datasets. MiRNAs are displayed on the X-axis, and odds ratio on the Y-axis. The odds ratios are calculated based on the overlap between the mTPs for a given miRNA detected by each strategy and the mTPs for the same miRNA in multiMiR. Only strategies showing a significant association for at least one correlation threshold (Fisher’s exact test $P < 0.05$) are shown. The number in black above each bar indicates the threshold, and the number in white inside the bar represents the total number of mTPs detected by the strategy for that miRNA that overlap with multiMiR.

Given the varying results for the different datasets in terms of optimal strategy and threshold, we further investigated how each miRNA responds to the different strategies. For this, we computed the specific odds ratio at the miRNA level for each strategy and threshold, based on the overlap between the mTPs detected for a given miRNA and the predicted/validated mTPs for the same miRNA in multiMiR. We then ranked the strategy and correlation threshold combinations based on this odd ratio and kept those that showed a significant overlap using Fisher's exact test. Applying the selection-integration strategy, we selected the top strategy and correlation threshold for each miRNA in each dataset as shown in Figure 3. There was no consensus best strategy for all miRNAs detected, with the exception of the LD dataset for which the CPM vs CPM strategy had the highest specific odds ratio for all DEMs, but there were still differences in optimal correlation threshold. Interestingly, analysis of both the LD and DCM datasets led to mTPs that included miR-155, and analysis of both the DCM and PMM2 datasets led to mTPs that included miR-183; however the optimal strategy differed between datasets. Other benchmarking measures such as specificity, accuracy, precision and recall were also calculated (Online Repository Files 8, 9 and 10: Section Tables ‘Best Strategy Benchmark’).

### Robustness to database changes

We investigated the robustness of the selection-integration method to potential changes in the underlying databases from which the multMiR mTPs were extracted. This was performed by comparing the ranking obtained using all multiMiR mTPs with the ranking from a randomized subset of 75%. This was repeated 40 times.

**Figure 4:**
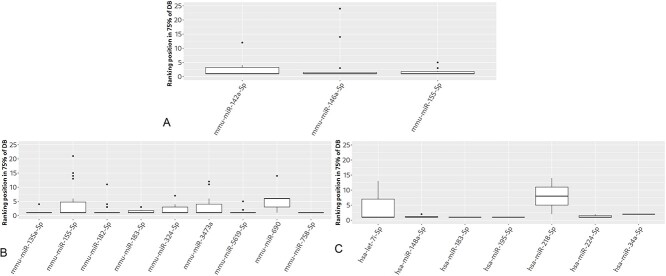
Rankings for the selection-integration method applied to randomized samples of multiMiR mTPs **A** LD, **B** DCM and **C** PMM2-CDG datasets. In some of the replicas, the top strategy was ranked relatively low when using the randomized subset, such as for miR-155, miR-324 and miR-3473a in the DCM dataset, and miR-let-7i in the PMM2-CDG dataset.

The top strategy obtained using all multiMiR mTPs was always top or close to top in the rankings obtained using the randomized subsets (Figure 4), with the exception of miR-218 and miR-34a in the PMM2-CDG dataset and miR-690 in the DCM dataset.

### Functional analysis of miRNA targets

We used the mTPs obtained using the selection-integration method that were also found in multiMiR to perform functional analysis. For each dataset, we looked for functional enrichment of GO terms and KEGG and Reactome pathways among the targets of each miRNA using over representation analysis.

#### Dilated cardiomyopathy

**Figure 5:**
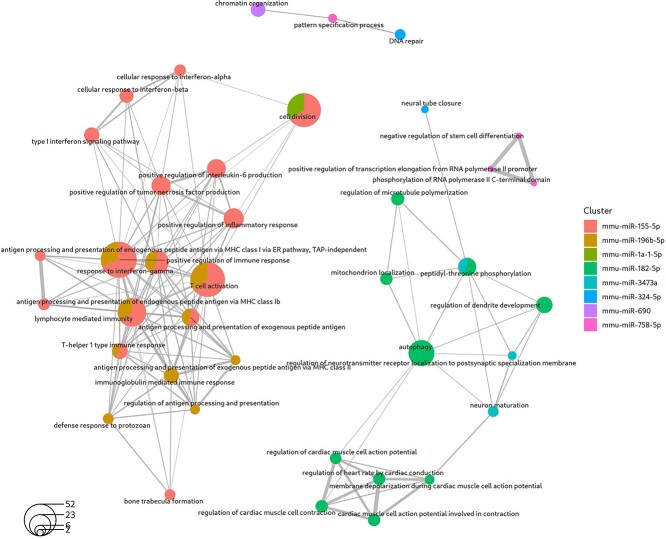
Top GO Biological Process terms enriched among the miRNA targets obtained from the mTPs found using the integrated strategy applied to the DCM disease dataset. Up to 10 significantly enriched terms are shown for each miRNA. The circles represent enriched GO terms, with the size of the circle corresponding to the number of genes among the targets that are annotated with that term (the transparent circle in the bottom left indicates the scale). The circles are colored according to the miRNA whose targets are annotated with that term. The circles with two colors represent terms that contain genes that are targets of two different miRNAs. The links between circles indicate that both terms contain the same gene.

Of the 53 DEMs identified in the DCM dataset, coRmiT found targets for nine (Figure 3B). Full details of the enriched categories among the targets of each of these miRNAs are shown for all annotation sources in Online Repository File 5. Enrichment results for GO Biological Process terms are shown in Figure 5. Interestingly, the targets of all nine miRNAs identified by coRmiT were enriched for GO Biological Processes, except for miR-183, which did not show enrichment for any term.

The top most significantly enriched terms for miR-155 and miR-196b targets were related to immune response regulation at different levels. The miR-196 targets are enriched in exogenous peptide antigen processing and presentation via MHC class II. On the other hand, miR-155 targets are enriched in the processing and presentation of endogenous peptide antigen through MHC class Ib via the ER and cell response/signaling for type I interferon (Figure 5).

The targets of miR-182 are also enriched in the modulation of heart rate by regulating cardiac conduction (Figure 5).

The targets of miR-690 and miR-758 are involved in DNA repair, chromatin organization and transcription regulation. Notably, the miR-758 targets involved in transcription regulation also exert a negative regulatory effect on the differentiation of stem cells (Figure 5).

#### PMM2-CDG

For the PMM2-CDG study, coRmiT found gene targets for seven miRNAs (Figure 3C). Detailed information on the enriched categories among the targets of each of these miRNAs are shown for all annotation sources in the Online Repository File 6.

**Figure 6:**
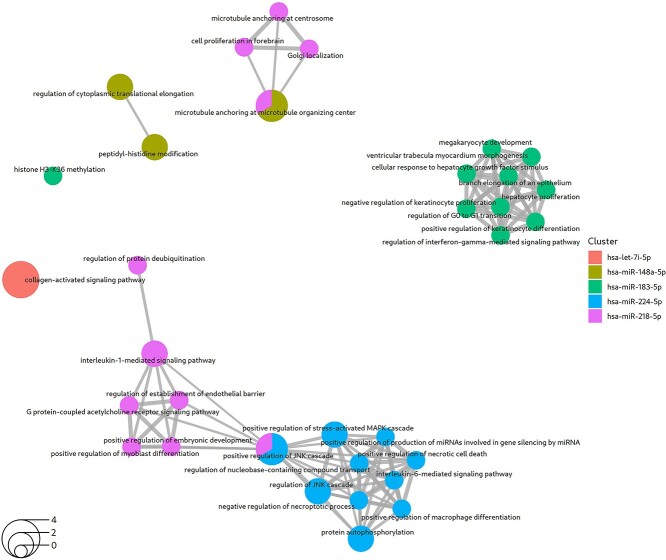
Top Reactome pathways enriched among the miRNA targets obtained from the mTPs found using the integrated strategy applied to the PMM2-CDG dataset. Up to 10 significantly enriched terms are shown for each miRNA. The circles represent enriched Reactome pathways, with the size of the circle corresponding to the number of genes among the targets that are annotated with that pathway (the transparent circle in the bottom left indicates the scale). The circles are colored according to the miRNA whose targets are annotated with that pathway. The circles with two colors represent pathways that contain genes that are targets of two different miRNAs. The links between circles indicate that both pathways contain the same gene.

The results for the Reactome and Biological Process subontologies are shown in Figure 6 and Online Repository File 6: Section BP Over Representation analysis, respectively. Enriched Reactome pathways were found for the targets of all seven miRNAs, except for miR-148a.

Enrichment in Biological Function terms was found for miR-let-7i, miR-224, miR-183, miR-218 and miR-148a. The only common term between targets of different miRNAs was the synthesis of IP3 and IP4 in the cytosol, which was also enriched among the targets of both miR-let-7i and miR-218.

Furthermore, miR-let-7i target genes were enriched in terms related to extracellular matrix components, specifically collagen, which confers tensile strength and activates tyrosine kinase receptors (Figure 6 and Online Repository File 6: Section BP Over Representation analysis).

The targets of miR-224 were enriched for necroptotic processes related to serine/threonine/tyrosin kinase activity, the Jun amino-terminal kinases (JNK) cascade, tumor necrosis factor receptors, cytokine-mediated signaling and protein autophosphorylation via the targeting of RIPK1 (Figure 6 and Online Repository File 6: Section BP Over Representation analysis).

#### Lafora disease

Regarding the LD dataset, coRmiT found significant targets for the three DEMs (Figure 3A). Full details of the enrichment analysis are shown in Online Repository File 4.

**Figure 7:**
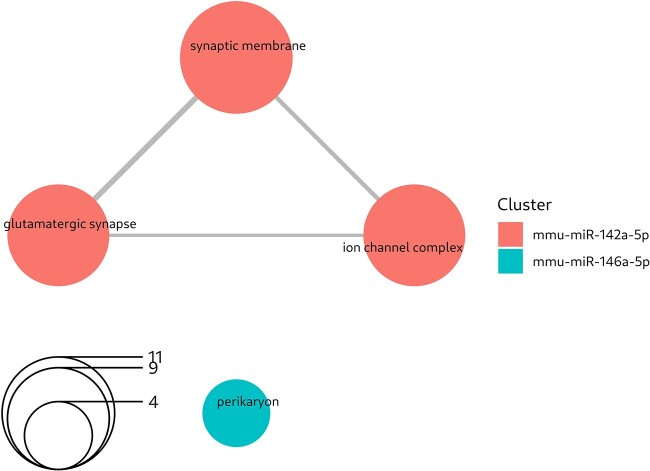
Top GO Cellular Component terms enriched among the miRNA targets obtained from the mTPs found using the integrated strategy applied to the LD dataset. The circles represent enriched GO terms, with the size of the circle corresponding to the number of genes among the targets that are annotated with that term (the transparent circle in the bottom left indicates the scale). Circles are colored according to miRNA whose targets are annotated with that term. The links between circles indicate that both pathways contain the same gene.

Focusing on the GO Cellular Components subontology, we found that miR-142a targets were enriched in terms related to the synaptic membrane, involved in the glutaminergic synapse and associated with the ion channel complex. In contrast, miR-146a targets are located in the perikaryon (Figure 7). Additionally, miR-155 targets are enriched in processes related to mRNA binding and transmitter-gated ion channel activity related processes (Online Repository File 4: Section BP Over Representation analysis).

**miR-155 also forms positively correlated mTPs**

We used coRmiT to find mTPs that showed positive correlation in the LD dataset, as explained in the Methods section. Only miR-155 mTPs showed significant overlap with multiMiR. The strategy and correlation threshold combination that led to the highest odds ratio was Cg Cm (normalized gene counts and normalized miRNA counts) with a correlation threshold of 0.9. The most significantly enriched terms and pathways for the targets of miR-155 were related to immune system activation, including cytokine activity, immunoglobulin and complement binding and apoptosis. Full details are given in Online Repository File 7.

**Validation of miRNA target expression changes**

**Figure 8:**
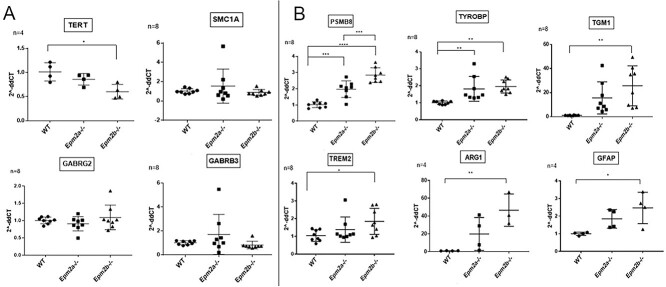
Analysis of the putative targets of miRNA in LD. **A** Includes anti-correlated targets and **B** includes the correlated targets. The graphs compare the 2-$\Delta \Delta{}CT$ values of wild-type samples, samples with the mutated laforin gene (*Epm2a-/-*) and the mutated malin gene (*Epm2b-/-*). Results are expressed as the mean $\pm $ SD. ^*^^*^^*^^*^P<0.0001, ^*^^*^^*^P<0.001, ^*^^*^P<0.01, ^*^P<0.05.

We analyzed the expression of the targets of miR-155 using RT-qPCR, for both correlated and anti-correlated mTPs. Four anti-correlated target genes were selected and are shown in Figure 8A. Among these, a significant change in expression could only be found for the *Tert* gene when comparing *Epm2b-/-* samples with wild-type. Additionally, six positively correlated targets were selected (Figure 8B). While *Tgm1*, *Trem2*, *Arg1* and *Gfap* genes were overexpressed in *Epm2b-/-* samples, both *Tyrobp* and *Psmb8* genes were overexpressed in *Epm2a-/-* and *Epm2b-/-* (Figure 8B).

## DISCUSSION

The use of anti-correlation between RNA-Seq and miRNA-Seq expression profiles is a commonly used technique to detect mTPs [17–20]. Multiple approaches based on different representations of gene expression profiles have been employed, however consensus on the optimal method remains unclear. Considering these premises, we have developed coRmiT, which implements multiple mTP detection strategies with varying correlation thresholds. We applied it to multiple datasets. Our results show that no single approach consistently outperforms others across all situations. We propose the selection of the strategy and threshold with the highest odds ratio for each miRNA, based on overlap with known and predicted mTPs, as a novel approach to combine strategies.

We applied our methodology to three rare disease datasets with different experimental designs, including differences in the organism studied and the number of samples. We applied the same expression analysis workflow upstream of coRmiT to ensure that the mTPs detected were not influenced unduly by aspects of the initial analysis [37]. It is noteworthy that the datasets had a relatively small number of samples per group, a common theme in rare disease analysis.

The datasets differed considerably in the number of DEGs, DEMs and co-expression modules detected, despite similar analysis settings (Table 1). Additionally, variations were observed in terms of the best strategy for detecting mTPs using the overall odds ratio. Specifically, the *Cg Cm*, *Eg Cm* (Eigengene of gene module and normalized miRNA counts) and *Eg Hm* (Eigengene of gene module and normalized expression of miRNA module hub gene) strategies yielded the highest odds ratios for the LD, DCM and PMM2-CDG datasets, respectively, at higher thresholds (Figure 2). We did not find any previous studies that used the correlation between miRNA modules and gene CPM, as such these strategies are not included here.

In addition to the differences observed in the expression results (Table 1), it is notable that strategies using individual miRNA profiles for detecting mTPs, rather than relying on miRNA co-expression modules, tended to perform best, in line with previous studies [17, 18].

In terms of the overall odds ratio, the PMM2-CDG dataset showed better results using a less strict threshold. This observation may be attributed to the distribution of correlation values between DEMs and genes, which appears to be shifted toward 0 in the PMM2 dataset compared with the others, as shown in the correlation distributions in Online Repository Files 8, 9 and 10. Consequently, this results in fewer correlated pairs at very strict thresholds, suggesting a weaker relationship between the expression of miRNAs and their targets. Possible explanations for this phenomenon include increased variability among the human samples or other characteristics related to the experimental design. These findings underscore the absence of an optimal strategy across all datasets. It is noteworthy that there is significant variance between datasets in terms of the overall odds ratios (Figure 2). This could be a consequence of combining the pairs of different miRNA to compute a unique measure, due to the varying numbers of mTPs for each miRNA in multiMiR. These differences were reduced when the odds ratio was computed specifically for each miRNA (Figure 3).

Regarding individual miRNAs, for both DCM and PMM2-CDG datasets the optimal strategy and threshold differed between miRNAs. In the case of the LD dataset, while the strategy remained consistent, the threshold differed (Figure 3). Interestingly, at the miRNA level, one of the strategies that performed poorly at the dataset level, Eg Em (Eigengene of gene module and eigengene of miRNA module), was the best for many of the miRNAs identified in the DCM dataset. Notably, we observed significant discrepancies between the specific odds ratio and the overall odds ratio when considering miRNAs individually, particularly for the LD dataset, for which the specific miRNA odds ratios were considerably smaller. This is because the overall odds ratio is sensitive to the number of mTPs for each miRNA in multiMiR.

To further explain this, we can use the LD dataset as an example: among the three DEMs detected, two (miR-155 and miR-146a) are highly represented in multiMiR. Consequently, when calculating the overall odds ratio, which considers all pair-wise permutations formed between expressed miRNAs and genes as the universe of pairs, the denominator ($S_{d}/R$ in the odds ratio formula in Supplementary Methods) is significantly smaller. This is due to the inclusion of many other miRNAs that are less well represented in multiMiR. Conversely, the specific miRNA odds ratio only includes the possible mTPs formed by the given miRNA and the expressed genes. In this case, the same miRNA is used in both the numerator and denominator of the odds ratio, mitigating bias introduced by differences in miRNA representation in the databases [38].

Moreover, when the same miRNA was detected in different datasets, we observed variation in the optimal strategy for detecting mTPs involving that specific miRNA. Collectively, these results suggest that each miRNA exhibits distinct regulatory behavior in terms of gene expression depending on the context, i.e., the disease or the tissue involved [39]. This aligns with our existing knowledge of miRNAs, where each miRNA has its own repression mechanisms and actions; a single or multiple miRNAs can inhibit the expression of multiple target genes or only a small number [40]. In cases where an miRNA acts on many targets, the repression tends to be milder [41]. These observations support the low correlation between the expression profile of an miRNA and their targets for certain strategies (Figure 3). These findings show that the optimal correlation strategy for identifying mTPs is likely to vary for each miRNA and differ in performance based on how well it reflects the underlying miRNA–mRNA regulation mechanisms.

This motivated the development of the selection-integration method, designed to treat each miRNA separately and select mTPs according to the strategy and threshold that leads to the greatest odds ratio. The other benchmarking measures showed differences in terms of how they ranked the different strategies. This reflects how these measures are calculated, being affected to different extents by both missing mTPs in multiMiR and conversely, mTPs in multiMiR that were not detected by coRmiT.

With the growing adoption of high-throughput techniques for mTP detection, like CLASH and Ago-CLIP, the amount of data in multiMiR and other databases will also grow. We therefore investigated the robustness of the selection-integration method to such changes. In simulation studies the top-performing strategy tends to remain unchanged for almost all miRNAs across iterations (Figure 4). Future work should look at other ways of integrating the results of the different strategies, taking into account the consensus between the results. Validation of the different integration methods should also be investigated.

Using the selection-integration method, we successfully detected mTPs for each dataset. We will now discuss the potential implications of these findings.

### Dilated cardiomyopathy

The functional enrichment analysis of the DCM dataset showed that the targets we detected for miR-155 play key roles in the regulation of the immune response. miR-155 has been shown to be key regulator of inflammation in different cardiovascular diseases, including DCM [42–46].

In accordance with our results, miR-155 was previously found to be overexpressed in cardiac tissue of inflammatory DCM patients. Notably, its expression levels correlated with inflammatory cell counts, supporting its role as an inflammatory marker in DCM [46]. Our analysis revealed that some of the miR-196b target genes encode structural constituents of the extracellular matrix contributing to tensile strength and participating in extracellular matrix binding. The accumulation of collagen and other components of the extracellular matrix, following cardiomyocyte death, cardiac fibrosis, is a key process in the progression of DCM [43].

Our results also suggest that miR-135a may be a potential regulator of the WNT pathway, a mechanism previously described as contributing to the pathophysiology of LMNA cardiomyopathy [31, 41]. Additionally, the targets of miR-182 showed enrichment for the cardiac conduction system. Abnormalities in this system are commonly observed for DCM associated with *LMNA* mutations [47], heightening the risk of arrhythmia.

The target genes of miR-324, miR-690 and miR-758 are involved in chromatin organization, a process known to be affected by mutations in the *LMNA* gene [48].

### PMM2-CDG

The application of coRmiT and the selection-integration method to the PMM2-CDG dataset revealed seven miRNA with targets exhibiting significant overlap with multiMiR mTPs. Notably, many targets of miR-let-7i are collagen type IV genes and are enriched for related processes as detailed in Online Repository File 6: Sections MF Over Representation Analysis, BP Over Representation Analysis and Reactome Over Representation Analysis. Previous studies have suggested that impairment of the collagen IV network contributes to PMM2-CDG-related symptoms, such as intra-cerebral hemorrhage and stroke-like episodes [29, 49, 50]. As such, we suggest further investigation into the role of collagen IV expression under the control of miR-let-7i in this disease.

Furthermore, our analysis revealed that miR-224 forms mTPs with TNF-$\alpha $ receptor genes. The activation of PMM2 activity with epalrestat has been recently shown to be related to the prevention of TNF-$\alpha $-mediated pro-inflammatory metabolism by increasing sensitization to the therapeutic effect of mannose [51]. Therefore, the miR-224 may have a role in regulating this process.

### Lafora disease

In the case of LD we found significant targets for all three DEMs, which showed over representation for GO terms related to the glutaminergic synapse and perikaryon (Figure 7 and Online Repository File 4: Section BP Over Representation Analysis).

We previously proposed the pharmacological modulation of glutaminergic and neuroinflammatory pathways as a therapeutic strategy [52]. In this study, we have shown that both miR-146a and miR-155 anti-correlated targets are related to glutaminergic receptor activity (Figure 7 and Online Repository File 4: Sections CC Over Representation Analysis and BP Over Representation Analysis).

Both miR-155 and miR-146a have been shown to be involved in complementary regulatory pathways activated during the inflammatory response, being the first pro-inflammatory and the second anti-inflammatory [53]. We showed that both miRNAs are overexpressed, in an age-dependent manner in brains from *Epm2a-/-* and *Epm2b-/-* mice models [30]. Interestingly, this overexpression coincided with the increased expression of putative target genes involved in the inflammatory cascade and oxidative stress responses, such as *Sod2*, *Socs1* and *Traf6* [30]. The joint action of both miRNAs has also been detected in systemic lupus erythematosus (SLE) patients [54]. Moreover, the overexpression of miR-155 and the insufficiency of Dicer may suggest a Dicer-independent alternative mechanism of miRNA regulation under inflammatory conditions in SLE [44].

Based on these results, we decided to extend our analysis toward the search of mTPs that showed a positive correlation between miRNAs and their target genes. Only miR-155 formed mTPs that were significantly over represented in multiMiR. We validated the differential expression of six of these targets using RT-qPCR in another set of samples. We were able to replicate all changes. Conversely, for the anti-correlated gene targets, we were able to replicate the differential expression results for one gene out of the four tested, *Tert* (Figure 8).

Functional analysis of the positively correlated target genes found roles in activating the inflammatory response and apoptosis (Online Repository File 7: Section BP Over Representation Analysis). Interestingly, we also detected underexpression of *Dicer1* in the LD RNA-Seq dataset (log2FC = -0.17 and FDR = 5.33e-06, Online Repository File 1). This may suggest that the previously described Dicer-independent mechanism of miRNA regulation detected in SLE is also present in LD. A relationship between the lack of malin in LD KO mice models and alterations in Dicer-mediated regulation of RNA expression has been reported previously [55].

In summary, the application of coRmiT to the three datasets, followed by functional analysis of the targets of detected mTPs, demonstrates the capability of our methodology to unveil molecular mechanisms potentially regulated by miRNAs and their impact on disease. Despite the insightful findings, our analysis faces limitations attributed to the limited information available in databases and the variable accuracy of mTPs prediction methods [38]. Another challenge is the over-representation of certain miRNAs in the databases, similar to observations for some genes [38]. This bias is expected to diminish as more data become available for a broader range of miRNAs. Additionally, our results highlight the robustness of coRmiT to changes in the underlying databases, as evidenced by the consistency in top-ranking strategies for each miRNA.

**Key Points**

- An integrated approach for miRNA–target detection based on correlation is needed
- We have implemented coRmiT to integrate the results of multiple strategies and select the optimal one
- The robustness of coRmiT to differences in the underlying validation dataset has been shown
- CoRmiT has been applied to three real case studies of rare diseases
- The discovered miRNA–target pairs are relevant to the diseases, and their expression has been validated

## Supplementary Material

Supplemantary_methods_bbae060

## Author Biographies

**José Córdoba-Caballero** is a PhD student at the Department of Molecular Biology and Biochemistry of University of Malaga and Biomedical Research and Innovation Institute of Cádiz, Spain. His research focuses on the development of bioinformatics software applied to the analysis of sequencing data, applied to disease.

**James R. Perkins** is a lecturer at the Department of Molecular Biology and Biochemistry, University of Malaga, Spain. He works on the development of software for the analysis of multi-omics data, applied to disease.

**Federico García Criado** is a PhD student at the Department of Molecular Biology and Biochemistry of University of Malaga. His research focuses on the development of bioinformatics tools for analyzing biological networks in rare diseases.

**Diana Gallego Martínez** is a PhD student at the Center of Molecular Biology Severo Ochoa, Spain. Her research is focused on characterizing the pathophysiology of inborn errors of metabolism to find novel therapeutic strategies.

**Alicia Navarro-Sánchez** is a biologist specialized in biomedical research. The results of this study are part of his master’s thesis during her stay in Faculty of Medicine and Dentistry in the University of Valencia, Spain.

**Mireia Moreno-Estelles** is a post-doctoral researcher in neuroscience at the CIBER of Rare Diseases (CIBERER), Instituto de Biomedicina del CSIC, Spain. Her research focuses on the role of astrocytes in Lafora Disease.

**Fernando Bonet** is a post-doctoral researcher in Molecular and Cellular Biology at the Department of Medicine, University of Cádiz, Spain. His research focuses on the emerging role of noncoding RNAs in heart diseases.

**Concepción Garcés** is a PhD student at the Department of Physiology, Faculty of Medicine and Dentistry, at the University of València, Spain. Her research focuses on the molecular mechanisms underlying inflammatory-related diseases, with the goal of identifying viable targets for the advancement of biomarker development.

**Carlos Romá-Mateo** is a staff professor and researcher at the Department of Physiology, Faculty of Medicine and Dentistry, at the University of València, Spain. His research focuses on the pathophysiology of inflammatory diseases such as sepsis and rare epileptic disorders.

**Rocio Toro** is a cardiologist and professor at the Department of Medicine, University of Cádiz, Spain. She leads a research group focused on genetic cardiovascular diseases and biomarkers

**Belen Pérez** is a professor of Biochemistry and Molecular Biology at the Autonomous University of Madrid, and a researcher at the Center of Molecular Biology. Her research is focused on improving the diagnosis of inborn errors of metabolism and developing therapeutic strategies.

**Pascual Sanz** is a Research Professor at the Institute of Biomedicine of Valencia (CSIC). He leads a research group interested in the study of the molecular basis of Lafora disease.

**Matthias Kohl** is a professor at the Faculty of Medical and Life Sciences, Furtwangen University, Germany. He heads a research group on data science for life science.

**Elena Rojano** is a post-doctoral researcher in bioinformatics at the Department of Molecular Biology and Biochemistry, University of Malaga, Spain. Her research focuses on systems biology methods for associating pathological phenotypes to genomic variants from patients with rare diseases.

**Pedro Seoane-Zonjic** is a post-doctoral researcher in bioinformatics at the CIBER of Rare Diseases (CIBERER), University of Malaga, Spain. His current research focuses on developing software for the analysis of rare diseases from high-throughput genomic data.

**Juan AG. Ranea** is a professor at the Department of Molecular Biology and Biochemistry, University of Malaga, Spain. He leads a research group focused on bioinformatics and systems biology for the analysis of rare diseases.
