# Supplementary material for: Exploring miRNA–target gene pair detection in disease with coRmiT
Source: Brief Bioinform. 2024 Mar 2;25(2):bbae060. doi: 10.1093/bib/bbae060 (PMC10939301; doi:10.1093/bib/bbae060)
Supplement: Supplemantary_methods_bbae060 [file supplemantary_methods_bbae060.pdf]

Supplementary Material

Supplementary Methods: This document explain the technical details about the computational procedure.

Additional results are available on the Online Repository: `https://github.com/JoseCorCab/coRmiT_additional_files`

Online Repository File 1: ExpHunterSuite results of Lafora Disease dataset.

Online Repository File 2: ExpHunterSuite results of Dilated Cardiomyopathy dataset.

Online Repository File 3: ExpHunterSuite results of PMM2-CDG dataset.

Online Repository File 4: GO, KEGG and REACTOME enrichments for targets found in Lafora Disease dataset.

Online Repository File 5: GO, KEGG and REACTOME enrichments for targets found in Dilated Cardiomyopathy dataset.

Online Repository File 6: GO, KEGG and REACTOME enrichments for targets found in PMM2-CDG dataset.

Online Repository File 7: GO, KEGG and REACTOME enrichments for the positive correlated targets of miR-155 found in Lafora Disease dataset.

Online Repository File 8: miRNA-target correlations analysis report using Lafora disease dataset.

Online Repository File 9: miRNA-target correlations analysis report using Dilated Cardiomyopathy dataset.

Online Repository File 10: miRNA-target correlations analysis report using PMM2-CDG dataset.

Online Repository Table 11: miRNA-target gene table that include the positively and negatively correlated mTPs found by coRmiT for the Lafora Disease dataset.

Supplementary Methods

miRNA-Seq and RNA-Seq expression workflow

miRNA detection

To obtain the miRNA data necessary to run coRmiT (Supplementary Figure 1A), smallRNA-Seq samples were first trimmed and cleaned using SeqtrimBB (`https://github.com/rafnunser/seqtrimbb.git`), an in-house tool based on BBmap [1], using a custom template for smallRNA-Seq which is included in the workflow code-base (`https://github.com/seoanezonjic/DEG_workflow`).

Preprocessed reads were mapped to the reference genome using Bowtie (v1.0) [2], using the options *–seedmms 0 –maqerr 80 –seedlen 10 –all -m 7 –best –strata*. Reads from the PMM2-CDG and DCM datasets were mapped to the human genome GRCh38; reads from the LD dataset were mapped to the mouse genome GRCm38. Similar mappings were combined following the miRDeep2 criteria using an in-house script collapse_bwt.rb, which is included in the workflow (`https://github.com/seoanezonjic/DEG_workflow`). Then, preprocessed reads and combined mapping files were analyzed with miRDeep2 [3] using known miRNAs for human or mouse, previously downloaded from the miRBase database, to predict existing and novel miRNAs for each sample, disabling the arguments for maximum number of precursors to analyze and the minimum read stack height thresholds using *-g -1 -a 1*. We selected all predicted miRNAs with a miRDeep2 score greater than 0. These options are the most sensitive possible: we used them to ensure that all potential miRNAs would be detected. We later implemented an additional step for miRNA quantification, using these predicted miRNAs. As such, miRNAs for which there was little evidence of expression were removed at this stage. We obtained the sequences of the precursors of the predicted miRNAs from the genome using a custom Ruby script. These sequences were merged to produce a fasta file. Redundancy was removed by merging similar sequences using CD-HIT-EST (v4.8.1) [4] with the option *-c 1*.

miRNA quantification and expression analysis

Preprocessed reads were mapped to the non redundant predicted miRNA precursors using Bowtie2 (v2.2.9) [5] with default options for single reads and were quantified using sam2counts [6](Supplementary Figure 1B). This resulted in a table of counts for all miRNAs across samples. This was analyzed by degenes_hunter.R [7] to obtain DEMs between case and control samples as indicated in the experimental design. The count matrices were normalized using the counts per million (CPM) method implemented by the edgeR [8] package and miRNAs with fewer than 2 CPM were filtered out. Four differential expression detection packages were executed: DESeq2 [9], edgeR [8], limma [10] and NOISeq [11]. Packages that did not find any miRNAs with absolute base 2 logarithmic fold change (log2FC) > 1 and False Discovery Rate (FDR) < 0.05, as calculated by each method, were removed. DEMs for downstream analysis where those detected by all remaining packages. The CPM table was used as input to the weighted gene correlation network analysis method (WGCNA) [12] to group miRNAs into co-expression modules.

The biweight midcorrelation metric was used to compute the correlation between miRNAs. Modules were defined using *deepSplit=3*, *mergecutHeight=0.15*, *detectcutHeight=0.995*, *minCoreKME=0.7* and *minCoreKMESize=20*.

RNA-Seq analysis

RNA-Seq samples were preprocessed with SeqtrimBB using the default template for transcriptomic data. Preprocessed reads were mapped to the genome and quantified using STAR (v2.5.3) [13] using the *–no-discordant –no-mixed* options for paired reads.

This resulted in tables of counts of genes per sample which were then analyzed using degenes_hunter.R [7] to detect differentially expressed genes (DEGs) between case and control samples. The table of counts was normalized using the CPM. DESeq2, edgeR, limma and NOISeq were executed; packages that did not find any expressed genes with absolute log2FC $>$ 1 and FDR < 0.05 were removed from the analysis. The DEGs were those genes detected by any package (in contrast to the DEMs which had to be detected by all packages). The CPM table was used to find co-expression modules using WGCNA as described above for the miRNA analysis.

Analyzing the correlation between miRNA and genes with coRmiT

Processing input

We calculated the Pearson correlation coefficient between genes and miRNAs based on the following criteria:

For genes, they must be DEG, or be included in a co-expression module that contains at least one DEG with a KME > 0.7, and the gene itself has a KME > 0.7 for that module. The KME measure, also known as module membership, is the Pearson correlation value between the gene expression profile and the module eigengene, defined as the first right-singular vector of the standardized module expression data [12, 14, 15].

For miRNAs, only DEMs were used. For each miRNA-/RNA module, we used either the eigengene or the hub gene as representative of the module. We defined the hub of a module as the gene/miRNA with the highest KME. The input of coRmiT can be filtered through the option *—tag_filter* and module membership threshold can be set by the option *–module_membership_cutoff*

Using single expression profiles and/or module representative profiles as input (eigengene profiles or hub gene expression profiles) we performed 7 correlation strategies using coRmiT that could be categorized into one three groups as showed in Table 1.

Table 1. Summary of the correlation strategies implemented in coRmiT. Strategies are classified according to the input data into three groups that combines CPM and co-expresison modules. The names of the strategies (column "Strategy name") are composed from the gene and miRNA profiles (columns "Gene profiles" and "miRNA profiles") that are used for correlation analysis. **CPM** means counts per million matrix, **eigengene** is the first right-singular vector of the standardized expression data of a module and **HUB** is the expression profile of the gene/miRNA with highest correlation with the module eigengene.

Strategy group	Gene profiles	miRNA profiles	Strategy name
gene CPM vs miRNA CPM	CPM	CPM	Cg Cm
gene modules vs miRNA CPM	HUB	CPM	Hg Cm
	eigengene	CPM	Eg Cm
gene modules vs miRNA modules	eigengene	eigengene	Eg Em
	eigengene	HUB	Eg Hm
	HUB	eigengene	Hg Em
	HUB	HUB	Hg Hm

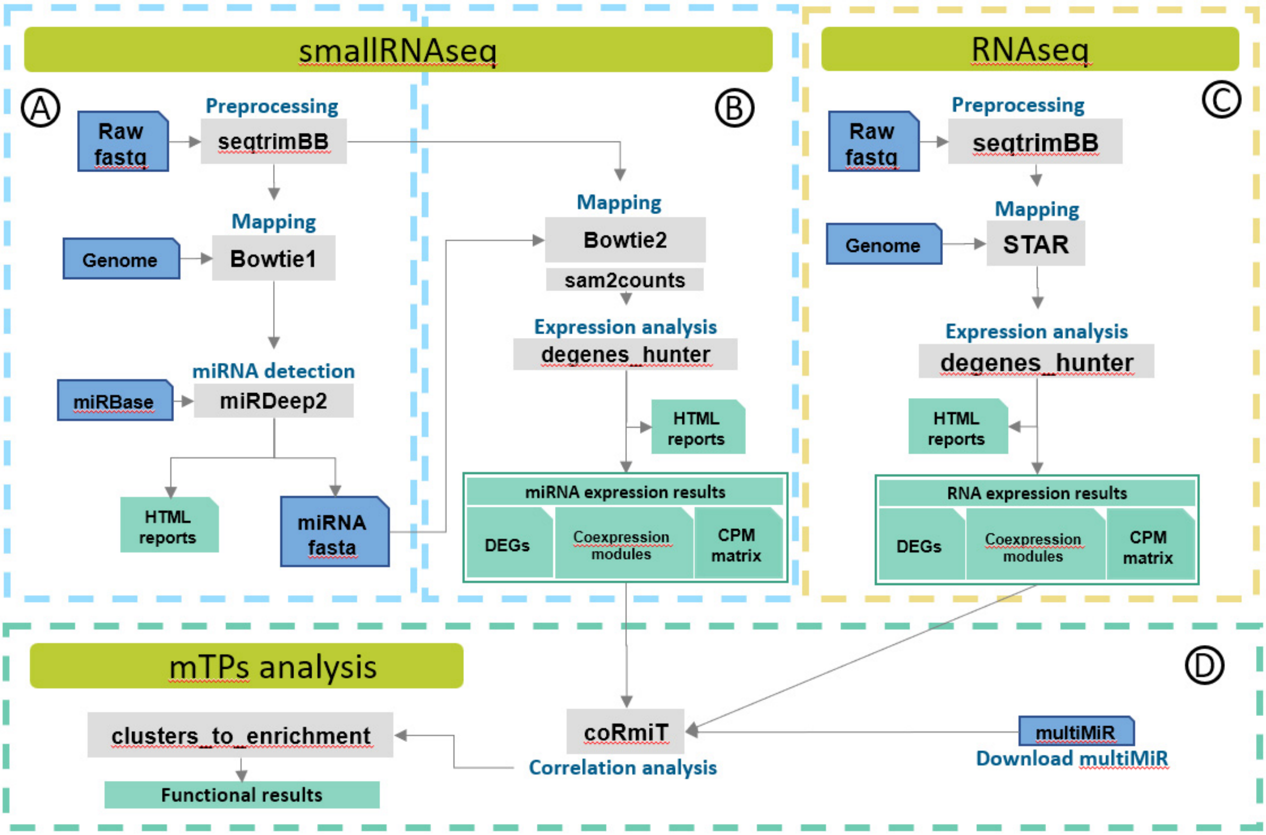

Supplementary Figure 1. General overview of the Expression analysis workflow. **A** miRNA detection using smallRNA-Seq samples; **B** miRNA quantification and expression analysis pipeline; **C** Gene expression analysis using RNA-Seq samples; **D** mTPs analysis using data obtained from the previous steps. **DEGs** and **DEMs** represents the differentially expressed genes and miRNAs respectively. **CPM matrix** is the expression matrix normalized to show counts per million mapped reads for each gene. **Eigen** and **hub** are the representative profiles of each co-expression module, i.e. the eigengene and the hub gene profiles. **mTPs** are the miRNA-target gene pairs

Comparing the performance of correlation strategies

Each of the strategies resulted in a list of predicted mTPs (strategy mTPs), which consisted of all miRNAs and target gene pairs with a correlation value lower than a given threshold. We then evaluated the results of all strategies obtained using a range of correlation thresholds, from -0.9 to -0.5 in intervals of 0.05. This was achieved by comparing the mTP lists for each strategy/threshold combination with experimentally validated and computationally predicted mTPs obtained from the multiMiR [16] meta-database, which integrates mTP data from eight predictive tools and three validation databases. The user can also load their own benchmark file of mTPs, such as mTPs predicted using deep learning methods like DMISO[17], TargetNet[18], miRBind[19] or ncRNAInter[20] using the option *–add_databases*.

We only used the mTPs included in multiMiR that existed in at least one validation database (multiMiR mTPs) and/or were predicted by at least two prediction tools to avoid false positives. Then, we compared strategy mTPs to the multiMiR mTPs to calculate the overall odds ratio for each strategy and threshold using the following equation:

$$\text{Odds ratio} = \frac{O/S_s}{S_d/R}$$

Where O represents the strategy mTPs that were found in the multiMiR mTPs and S_s represents those that were not, S_d represents possible permutations between expressed miRNA and genes that were found in the multiMiR mTPs but were not found by the strategy and R the possible permutations between expressed miRNA and genes that did not appear in either strategy or multiMiR mTPs.

Selecting the optimal mTPs for each miRNA to integrate the results of the different strategies

We have designed a selection-integration method that, for each miRNA, suggests potential mTPs from the data by using the results of the best peforming strategy and threshold.

For this, we computed a miRNA specific odds ratio for each DEM, comparing the mTPs found for each strategy and correlation threshold with the multiMiR mTPs. The miRNA specific odds ratio was calculated with the same formula as the overall odds ratio mentioned previously. The one-tailed Fisher's exact test was used as a significace measure, along with the odds ratio. The significant strategy/correlation threshold combinations were those that led to a p-value < 0.05 and odds ratio higher than 1.

For the selection-integration method, for each miRNA, we ranked the significant strategies and correlation threshold combinations by their odds ratios. When multiple strategies had the same odds ratio, we ranked more highly the strategy with the more restrictive correlation threshold the higher number of pairs that were multiMiR mTPs. The mTPs from the top

ranked strategy and correlation threshold combination for each miRNA were selected.

Validation of differential expression of targets in Lafora Disease

The expression levels of the putative LD targets were analyzed by RT-qPCR to confirm differential expression. cDNA was prepared from total RNA extracts using the High-Capacity cDNA Reverse Transcription Kit (Applied Biosystems, Life Technologies, Foster City, CA, USA) following the manufacturer's recommendations. We adapted the calculations for introducing 200 ng/μL of RNA in a 20 μL reaction volume. For a single sample, the master mix preparation comprised 10 μL of RNA, 2 μL of pool RT primers (5x), 0.8 μL of dNTPs (100 mM), 1 μL of MultiScribe reverse transcriptase (50 U/μL), 2 μL of 10X reverse transcription buffer, and 4.2 μL of nuclease-free water. A negative control of nuclease-free water was included to ensure no contamination with genomic DNA. The parameters for thermal cycling were 10 min at 25 °C, 120 min at 37 °C, and 5 min at 85 °C. For the real-time quantitative PCR, three technical replicates of each cDNA sample were generated for each gene to be tested, all in a 384-well plate using a QuantStudio 5 Real-Time PCR System thermocycler (Thermo Fisher Scientific). For each 10 μL of master mix, 1 μL of cDNA and 3.5 μL of nuclease-free water was mixed with 0.5 μL TaqMan® Gene Expression Assay (20x) (specific for each gene: Mm00436931_m1 for *Tert*, Mm00498375_m1 for *Tgm1*, Mm04209424_g1 for *Trem2*, Mm00490624_m1 for *Smc1a*, Mm00433489_m1 for *Gabrg2*, Mm00433473_m1 for *Gabrb3*, Mm01253033_m1 for *Gfap*, Mm00449152_m1 *Tyrobp*, Mm00475988_m1 for *Arg1*, Mm00440207_m1 for *Psmb8*) and 5 μL of TaqMan Gene Expression Master Mix (Applied Biosystems). The reactions were carried out using the following parameters: 50 °C for 2 min, 95 °C for 10 min, 40 cycles of 95 °C for 15 s, and 60 °C for 1 min. Finally, all RT-qPCR data were captured using the QuantStudio™ Design and Analysis Software (version 1.5.1, Thermo Fisher). The relative expression of each sample was calculated using the 2-$\Delta\Delta CT$ method (Livak & Schmittgen, 2001), using the CT (cycle threshold) value of *Gapdh* (Mm99999915_g1) as an internal control for the normalization of the CT of each sample. Graphical representations and statistical analyzes were conducted using GraphPad Prism v.8.3.0 for Windows (GraphPad Software, San Diego, CA, USA, www.graphpad.com). The selection of the targets was performed manually according their potential key role regulating the LD behaviour from the Online Repository Table 11.

References

1. B. Bushnell. BBmap Suite, 2014. URL `https://sourceforge.net/projects/bbmap/`.
2. Ben Langmead, Cole Trapnell, Mihai Pop, and Steven L. Salzberg. Ultrafast and memory-efficient alignment of short DNA sequences to the human genome. *Genome Biology*, 10(3):R25, March 2009. ISSN 1474-760X. doi: 10.1186/gb-2009-10-3-r25. URL `https://doi.org/10.1186/gb-2009-10-3-r25`.
3. Marc R. Friedländer, Sebastian D. Mackowiak, Na Li, Wei Chen, and Nikolaus Rajewsky. miRDeep2 accurately identifies known and hundreds of novel microRNA genes in seven animal clades. *Nucleic Acids Research*, 40(1):37–52, January 2012. ISSN 1362-4962. doi: 10.1093/nar/gkr688.
4. Limin Fu, Beifang Niu, Zhengwei Zhu, Sitao Wu, and Weizhong Li. CD-HIT: accelerated for clustering the next-generation sequencing data. *Bioinformatics (Oxford, England)*, 28(23):3150–3152, December 2012. ISSN 1367-4811. doi: 10.1093/bioinformatics/bts565.
5. Ben Langmead and Steven L. Salzberg. Fast gapped-read alignment with Bowtie 2. *Nature Methods*, 9(4):357–359, March 2012. ISSN 1548-7105. doi: 10.1038/nmeth.1923.
6. V Buffalo. sam2counts, 2010.
7. Fernando M. Jabato, José Córdoba-Caballero, Elena Rojano, Carlos Romá-Mateo, Pascual Sanz, Belén Pérez, Diana Gallego, Pedro Seoane, Juan A. G. Ranea, and James R. Perkins. Gene expression analysis method integration and co-expression module detection applied to rare glucide metabolism disorders using ExpHunterSuite. *Scientific Reports*, 11(1):15062, July 2021. ISSN 2045-2322. doi: 10.1038/s41598-021-94343-w.
8. Yunshun Chen, Aaron T. L. Lun, and Gordon K. Smyth. From reads to genes to pathways: differential expression analysis of RNA-Seq experiments using Rsubread and the edgeR quasi-likelihood pipeline. *F1000Research*, 5:1438, 2016. ISSN 2046-1402. doi: 10.12688/f1000research.8987.2.
9. Michael I. Love, Wolfgang Huber, and Simon Anders. Moderated estimation of fold change and dispersion for RNA-seq data with DESeq2. *Genome Biology*, 15(12):550, 2014. ISSN 1474-760X. doi: 10.1186/s13059-014-0550-8.
10. Matthew E. Ritchie, Belinda Phipson, Di Wu, Yifang Hu, Charity W. Law, Wei Shi, and Gordon K. Smyth. limma powers differential expression analyses for RNA-sequencing and microarray studies. *Nucleic Acids Research*, 43(7):e47, April 2015. ISSN 1362-4962. doi: 10.1093/nar/gkv007.
11. Sonia Tarazona, Pedro Furió-Tarí, David Turrà, Antonio Di Pietro, María José Nueda, Alberto Ferrer, and Ana Conesa. Data quality aware analysis of differential expression in RNA-seq with NOISeq R/Bioc package. *Nucleic Acids Research*, 43(21):e140, December 2015. ISSN 1362-4962. doi: 10.1093/nar/gkv711.
12. Peter Langfelder and Steve Horvath. WGCNA: an R package for weighted correlation network analysis. *BMC bioinformatics*, 9:559, December 2008. ISSN 1471-2105. doi: 10.1186/1471-2105-9-559.
13. Alexander Dobin, Carrie A. Davis, Felix Schlesinger, Jorg Drenkow, Chris Zaleski, Sonali Jha, Philippe Batut, Mark Chaisson, and Thomas R. Gingeras. STAR: ultrafast universal RNA-seq aligner. *Bioinformatics (Oxford, England)*, 29(1):15–21, January 2013. ISSN 1367-4811. doi: 10.1093/bioinformatics/bts635.
14. Peter Langfelder and Steve Horvath. Eigengene networks for studying the relationships between co-expression modules. *BMC systems biology*, 1:54, November 2007. ISSN 1752-0509. doi: 10.1186/1752-0509-1-54.
15. Lin Song, Peter Langfelder, and Steve Horvath. Comparison of co-expression measures: mutual information, correlation, and model based indices. *BMC bioinformatics*, 13:328, December 2012. ISSN 1471-2105. doi: 10.1186/1471-2105-13-328.
16. Yuanbin Ru, Katerina J. Kechris, Boris Tabakoff, Paula Hoffman, Richard A. Radcliffe, Russell Bowler, Spencer Mahaffey, Simona Rossi, George A. Calin, Lynne Bemis, and Dan Theodorescu. The multiMiR R package and database: integration of microRNA-target interactions along with their disease and drug associations. *Nucleic Acids Research*, 42(17):e133, 2014. ISSN 1362-4962. doi: 10.1093/nar/gku631.

17. Amlan Talukder, Wencai Zhang, Xiaoman Li, and Haiyan Hu. A deep learning method for miRNA/isomiR target detection. *Scientific Reports*, 12(1):10618, June 2022. ISSN 2045-2322. doi: 10.1038/s41598-022-14890-8.
18. Seonwoo Min, Byunghan Lee, and Sungroh Yoon. TargetNet: functional microRNA target prediction with deep neural networks. *Bioinformatics (Oxford, England)*, 38 (3):671–677, January 2022. ISSN 1367-4811. doi: 10.1093/bioinformatics/btab733.
19. Eva Klimentová, Václav Hejret, Ján Krčmář, Katarína Grešová, Ilektra-Chara Giassa, and Panagiotis Alexiou. miRBind: A Deep Learning Method for miRNA Binding Classification. *Genes*, 13(12):2323, December 2022. ISSN 2073-4425. doi: 10.3390/genes13122323.
20. Hanyu Zhang, Yunxia Wang, Ziqi Pan, Xiuna Sun, Minjie Mou, Bing Zhang, Zhaorong Li, Honglin Li, and Feng Zhu. ncRNAInter: a novel strategy based on graph neural network to discover interactions between lncRNA and miRNA. *Briefings in Bioinformatics*, 23(6):bbac411, November 2022. ISSN 1477-4054. doi: 10.1093/bib/bbac411.
